# Private Hospital for the Insane at Chicago

**Published:** 1847-08

**Authors:** 


ARTICLE IV.

### PRIVATE HOSPITAL FOR THE INSANE AT CHICAGO.

Dr. Edward Mead, late of St. Charles, Illinois, has resigned his place in the medical department of Illinois College, at Jacksonville, and removed to Chicago, for the purpose of opening a private hospital for the insane. For immediate use he had taken a private dwelling where the patients may receive better care than at their own homes, and already has several cases upon his hands which were so urgent as not to admit of delay in treatment.

He has procured, in the vicinity of the city, twenty acres of ground, favorably situated, upon which he will proceed to erect suitable buildings for permanent use. This will be ready for the reception of patients during the approaching autumn. There is no hospital for insane persons, in operation, within several hundred miles of Chicago, and none in any part of the United States not already filled to overflowing, so that the establishment of this seems a matter of urgent necessity.

Dr. Mead has devoted much attention to the subject, and will have such attendants for his patients as will ensure their good treatment and comfort. The terms, we hear, will be four dollars per week; or, with an especial attendant, ten dollars per week. D. B.

